# Helping People Hear Better with “Smart” Hearing Devices

**Published:** 2022-04-25

**Authors:** Tobias Goehring, Jessica Monaghan

**Affiliations:** 1 Cambridge Hearing Group, MRC Cognition and Brain Sciences Unit, University of Cambridge, Cambridge, United Kingdom; 2 National Acoustic Laboratories, Sydney, NSW, Australia

## Abstract

Millions of people around the world have difficulty hearing. Hearing aids and cochlear implants help people hear better, especially in quiet places. Unfortunately, these devices do not always help in noisy situations like busy classrooms or restaurants. This means that a person with hearing loss may struggle to follow a conversation with friends or family and may avoid going out. We used methods from the field of artificial intelligence to develop “smart” hearing aids and cochlear implants that can get rid of background noise. We play many different sounds into a computer program, which learns to pick out the speech sounds and filter out unwanted background noises. Once the computer program has been trained, it is then tested on new examples of noisy speech and can be incorporated into hearing aids or cochlear implants. These “smart” approaches can help people with hearing loss understand speech better in noisy situations.

## Hearing and Hearing Loss

Sound is created when objects vibrate. These vibrations travel through the air as sound waves. When sound waves reach our ears, they travel from the outer ear, down a tube called the ear canal, then they vibrate the eardrum and tiny bones in the middle ear, to reach the inner ear or cochlea. In the cochlea, thousands of sensory hair cells translate sound vibrations into electrical signals that then travel along nerves to the brain. The brain receives these signals and perceives them as speech, music, or other sounds.

**Cochlea**

The spiral-shaped part of the inner ear that contains the sensory organ of hearing.

Around one in five people in the world has some degree of hearing loss. This adds up to around 1.5 billion people, so hearing loss is a huge problem with societal, health, and economic impact. The World Health Organization predicts numbers will rise to one in four people by 2050. Currently, 400,000,000 people have hearing loss serious enough to require hearing aids. There are many different reasons why hearing loss occurs, for example getting older, listening to too much very loud music, or due to illnesses or medicines that harm the ears. The most common type of hearing loss affects important processes in the inner ear that convert sound waves into nerve signals and this can cause difficulties understanding speech.

## Devices for People with Hearing Loss

**Figure 1:**
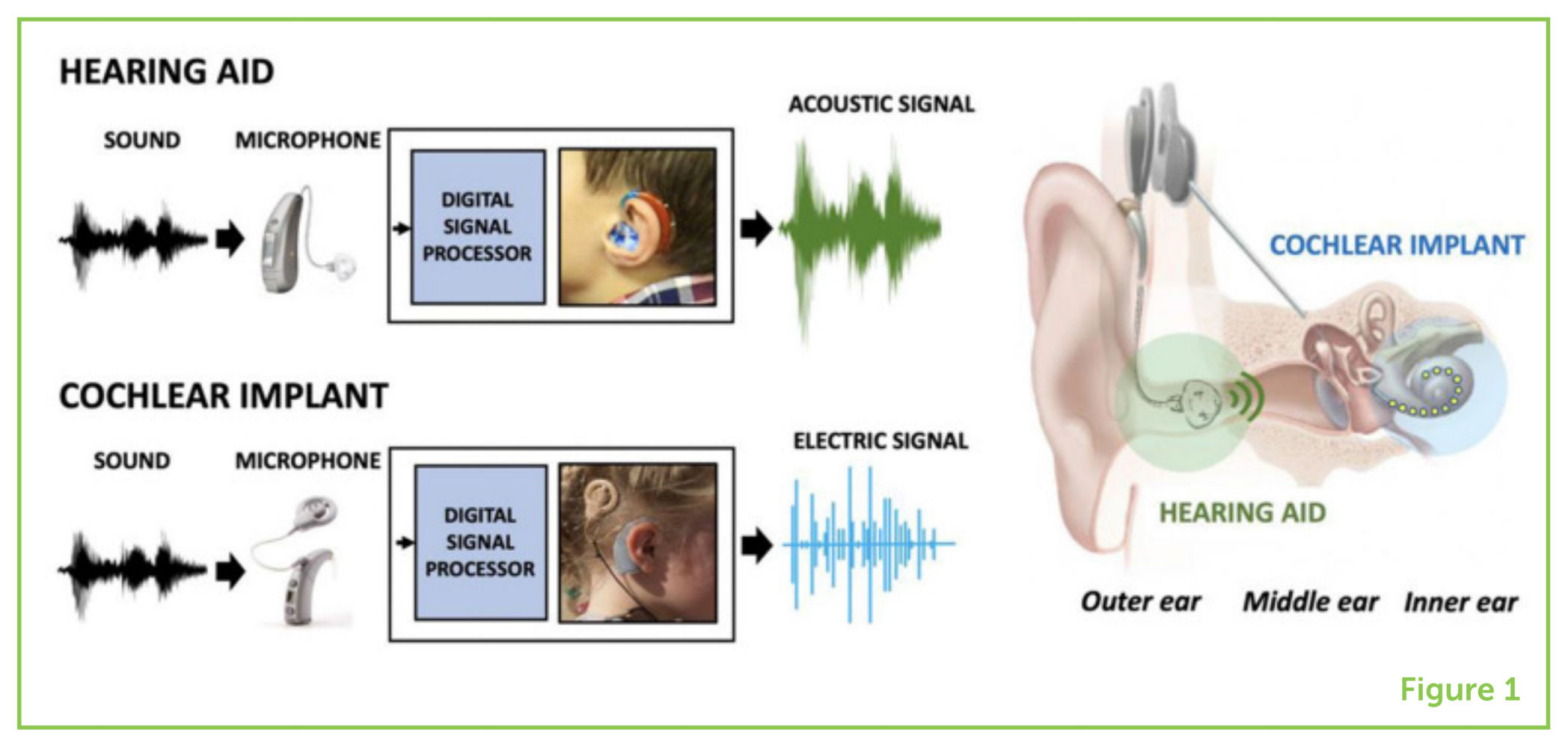
Both hearing aids and cochlear implants record sound with a microphone and process it with a digital signal processor, to optimize the sound for the person’s hearing loss. The hearing aid then uses a small loudspeaker in the ear canal to present the amplified sound as an acoustic signal (green). The cochlear implant uses tiny electrodes inside the cochlea to present the sound as an electric signal (blue). Depending on the degree and type of hearing loss, either a hearing aid or a cochlear implant (or a combination of both) can be used to help a person hear better.

Certain devices can help people with hearing loss. Depending on the amount of hearing loss, two main types of hearing devices are available. For most forms of hearing loss, acoustic hearing aids work well. These have a tiny microphone that changes sound into an electrical signal that is sent to a digital signal processor—a device that can very quickly modify sounds and amplify them (make them louder), to make sounds easier for the person to hear (Figure 1). The louder, modified sound is carefully optimized to suit the specific needs of the person before it is played into the person’s ear using a small loudspeaker positioned by the ear canal.

**Acoustic Hearing Aids**

A small electro-acoustic device to let people hear better.

**Digital Signal Processor**

A small computer chip used in many electronic devices to process signals such as speech, sound, video, or other digital data.

If hearing loss is severe, acoustic hearing aids may not provide enough amplification. In these cases, cochlear implants can be used. Cochlear implants also have an external microphone and a digital signal processor, but they also have an internal part, consisting of tiny electrodes, that is implanted inside a person’s cochlea by a surgeon. The internal part of the cochlear implant turns the sound information into electrical signals (Figure 1). The sound is split into different frequencies, ranging from low to high tones. Higher tones stimulate electrodes at the beginning of the cochlea and lower tones stimulate electrodes further into the cochlea. It is important to match the electrical signal to the location of the nerves along the cochlea so that the person can hear different tones and hear them in the correct order, from low to high.

**Cochlear Implants**

A small electronic device to let people hear via electrical stimulation of the auditory nerve in the cochlea.

**Frequencies**

The rate at which a sound wave vibrates. Lower frequencies (slower vibrations) are heard as lower tones (like a man’s voice); higher frequencies are heard as higher tones (like a child’s voice).

## People with Hearing Loss Struggle to Hear Well in Noisy Places

Even when using a hearing aid or a cochlear implant, it is often much more difficult for people to understand speech in noisy situations such as a busy classroom, workplace or restaurant. This means that a person with hearing loss might struggle to understand what friends or family are saying to them and may not want to take part in conversations.

Difficulty hearing speech happens because hearing aids and cochlear implants cannot recover the fine details of sound that are needed to understand speech, so the sound information that reaches the brain is not as detailed as it needs to be. People with hearing devices may still find it difficult to hear pitch (how high or low a tone is) or whether a person’s voice rises at the end of a sentence to form a question. Some people with hearing aids or cochlear implants also find it hard to tell different sounds apart. This makes it particularly difficult for them to follow and to understand speech correctly when the background is noisy. Even the best hearing aids and cochlear implants cannot get rid of background noise in real-life situations.

## Smart Computer Programs that Learn From their Mistakes

We are developing smart algorithms (computer programs) that can learn to make speech in noisy situations clearer and easier to understand. An algorithm is a set of rules followed by a computer, to perform a task or to solve a problem.

**Algorithms**

A set of rules to be followed for a process or operation, for example by a computer.

In previous research, speech was separated from noisy background sounds based on fixed rules, similar to a recipe. These rules had to be followed strictly by the digital signal processor in the hearing device.

This method only works for background sounds that are very different from speech. But in the real world, background noise often contains speech-like sounds, such as people chatting. For these situations, fixed rules do not work well. In the field of artificial intelligence, the goal is to build machines or computer programs that can learn from data. Researchers have begun to use these techniques to improve hearing aids and cochlear implants.

**Artificial Intelligence**

Methods to enable computers and algorithms to perform some intellectual tasks such as decision making, problem solving, communication, or perception.

**Figure 2:**
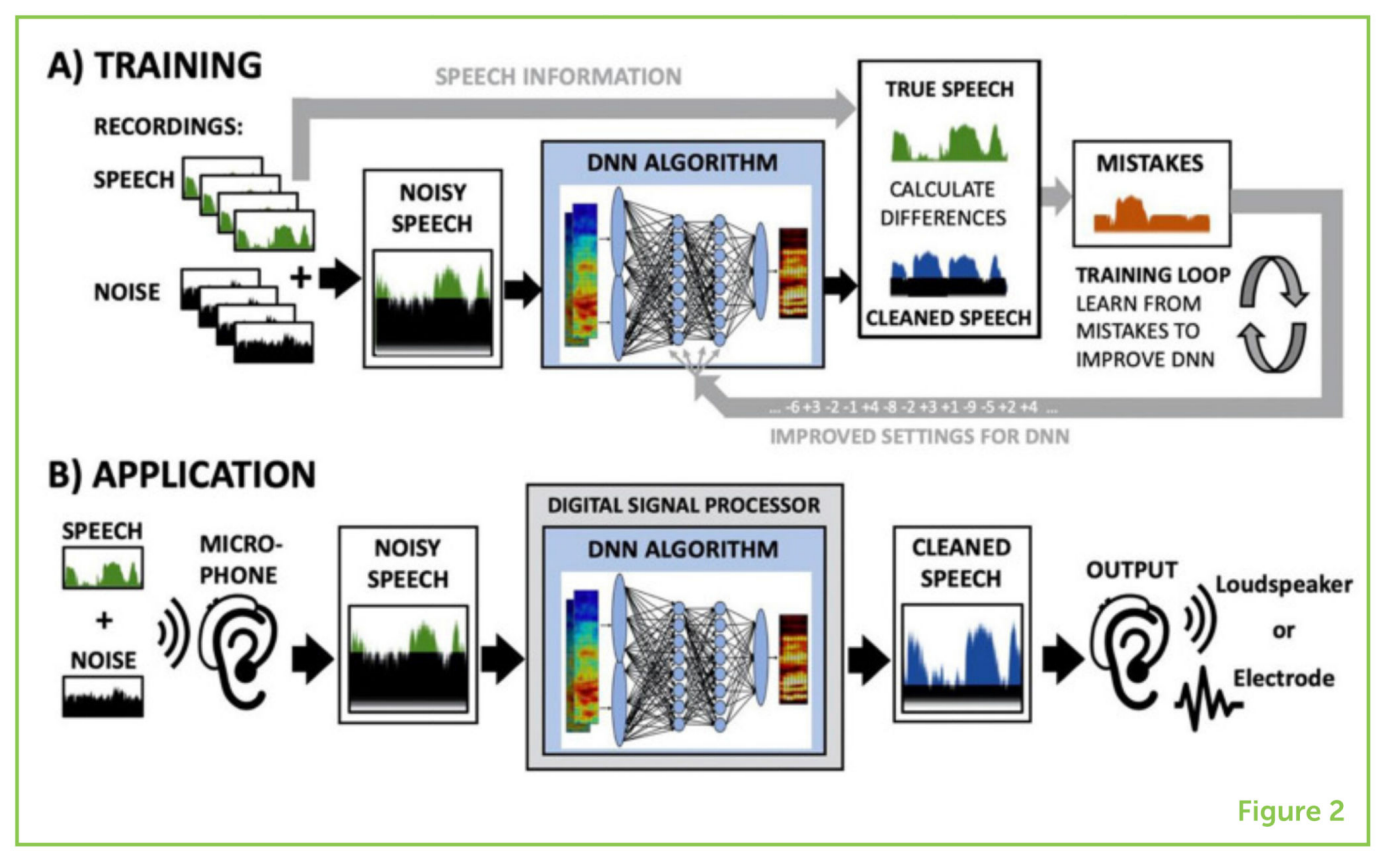
**(A)** Many examples of speech and noise recordings are mixed together to get noisy speech. The DNN algorithm then tries to clean up the speech, which is compared against the true speech to calculate the algorithm’s mistakes. Over many thousands of training loops, the DNN algorithm is adjusted step-by-step, based on learning from its mistakes, to get better at cleaning up the noisy speech. **(B)** After training is complete, the DNN algorithm can then be used in the digital signal processor of hearing aids or cochlear implants.

The specific algorithm we used is called an artificial deep neural network (DNN), which simulates the way the human brain learns. Because the human brain is very complex and powerful, DNN algorithms are not nearly as smart, but they are still much more powerful than older algorithms built on fixed rules. DNNs learn a set of rules depending on the task. In our research, we train DNNs to learn how to improve how speech sounds when there is background noise. To do this, we record lots of people saying a range of sentences. We then add realistic background noise, recorded in a busy restaurant or beside a busy road, and we play the mixed sounds to the DNN. We do not provide a set of rules to the DNN, but we tell the DNN which sounds make up the speech and which sounds make up the noise to get rid of. We basically teach the DNN step-by-step how to perform this task (Figure 2A).

**Artificial Deep Neural Network**

A computer algorithm inspired by the neural networks found in biological brains.

At the beginning of the training, the DNN makes a lot of mistakes because it has no experience in cleaning up speech signals. But these mistakes are important and valuable, because we can use them to teach the DNN how to make decisions about which sounds to keep and which sounds to filter out. During training, the DNN builds its own set of rules, by adjusting the settings of its parameters step-by-step. By listening to thousands or even millions of examples of many kinds of noisy speech mixes, the DNN’s parameters are optimized to reduce the number of mistakes it makes when cleaning up speech. Once training is complete, the DNN algorithm can be added to the digital signal processors of hearing aids and cochlear implants (Figure 2B).

In our research, we trained DNNs to improve noisy speech as much as possible and then performed listening tests to study how well the algorithm worked for people that use hearing aids [1] and cochlear implants [2, 3]. We measured how easy it was for our test subjects to hear the words in sentences, by counting the words they understand correctly, and we measured how pleasant the sounds were to hear, by asking them for their opinions.

## Results From the Listening Tests

**Figure 3:**
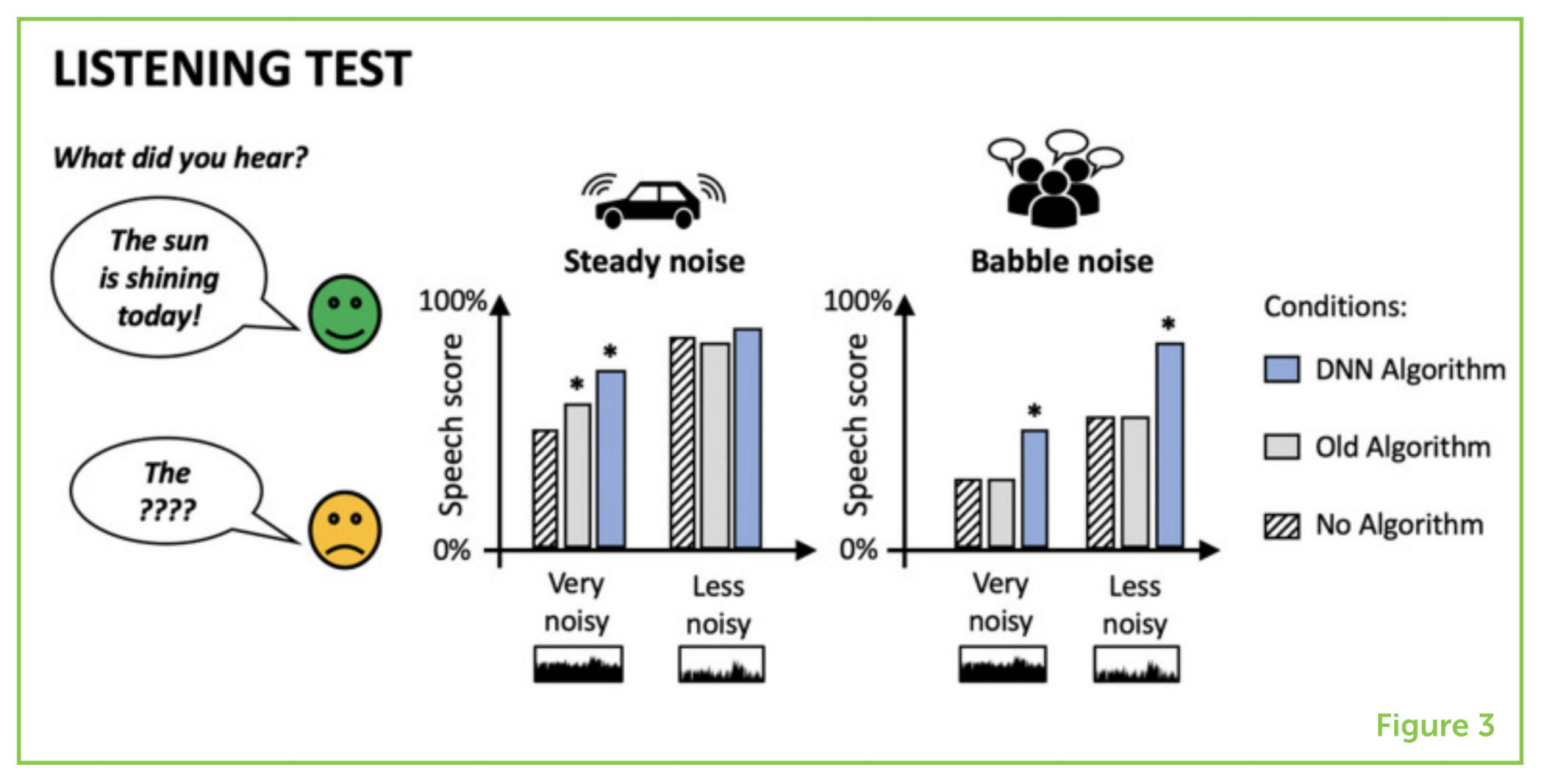
People with hearing devices listened to noisy sentences that were cleaned up by the DNN algorithm, an old, fixed-rules, algorithm, or no algorithm. The more words that were understood correctly, the higher the speech score. Speech was mixed with either steady background noise or “babble” noise, like many people talking. Very noisy and less noisy situations were tested. Asterisks mean that the result is probably real—not based on luck. Results show that the DNN algorithm improved speech scores more than the old algorithm did, especially with babble noise.

We found that the DNNs improved the understandability of speech by up to 14% for people using hearing aids (Figure 3) [1] and by about 30–40% for people using cochlear implants [2, 3]. The DNN algorithm also worked better than the fixed-rules algorithms used in the past.

Even a small amount of background noise causes difficulties for people with cochlear implants. So, the DNN algorithms we are building sometimes work better for people with cochlear implants than those with hearing aids because the algorithms do not need to filter out as much noise to make a difference. The DNN algorithms also work better if there is less noise to remove. Overall, the DNN algorithms perform well when speech is at least as loud as background noise, but they start to struggle when the noise is louder than the speech. DNNs successfully improved speech scores even when there were many people talking in the background, which is a clear improvement over previous algorithms, which did not work well in those cases.

We also found that it is much easier for the DNN algorithm to improve speech signals when it has been trained on a particular voice or a specific background noise; it does not perform as well for completely different speech and noise recordings that were not part of the training. This can be seen as a strength of DNN algorithms, because we can fine-tune them to work better with specific voices or noises. However, it can also be seen as a weakness because the DNN algorithm may become too specialized and then would not help much in other listening situations, for example with other voices.

## Summary

For many people who have hearing aids or cochlear implants, listening to speech in noisy situations can be difficult and tiring. We are building smart algorithms based on artificial intelligence techniques that can be used to improve future hearing aids and cochlear implants, particularly to help get rid of background noises. The algorithms use artificial DNNs that become smarter by learning from their own mistakes during training with many thousands of noisy speech examples. These algorithms are then used to clean up speech recordings before presenting those recordings to people with hearing loss and measuring their speech scores. Our results showed DNNs resulted in improvements in how well people could hear speech in the presence of noise, and how much they liked the sound of that speech. These results, along with other studies [4, 5], show that there is great potential to improve hearing aids and cochlear implants. With “smart” technology and algorithms such as DNNs, we can help people with hearing loss to hear speech better and thus help to make their communication easier.
